# A New Process for Preserving Meats and Liquids

**Published:** 1888-06

**Authors:** 


### A NEW PROCESS FOR PRESERVING MEATS AND LIQUIDS.

The problem of the preservation of liquids, such as new ale, draught beer, cider, grape juices, fruit syrups, and milk in their normal condition, has been the subject of numerous experiments extending over many years, and involving the expenditure of large sums of money. The final development of the invention in question has been largely due to the perseverence of Mr. W. B. Murdoch, the President of the company.

"It is claimed that the new process, which, briefly speaking, consists in first exhausting all the air from the fluid and then substituting in its place carbonic acid gas, will recommend itself to brewers, bottlers, dairymen, farmers, and fruit preservers, to whom it will prove of inestimable value. It is hardly too much to say that it will revolutionize the bottling industry by arresting fermentation, doing away with the old method of steaming and cooking, and avoiding the waste and breakage of bottles, which at present are the main objections to bottled liquids, especially beer and ale.

"It is a well-known fact that the oxygen of the air is the most essential element for the support, and maintenance of life, whether vegetable or animal, and when this element has been exhausted or removed from any medium or enclosure life of every kind becomes extinct. It is also a well-established fact that decomposition or fermentation is due to and carried on by the growth and development of the lower forms of life, which organisms are capable of extracting from the different fluids in which they multiply sufficient oxygen for their support, If, therefore, the oxygen contained in the various liquids to be preserved is entirely eliminated by some efficient mechanical process and then a pure antiseptic gas introduced to take its place, the fluids prepared in this manner will keep perfectly sweet and preserve their normal condition.

'This," he continued, "is precisely what is accomplished by the process of the company, and it is in this condition that cider, wines, beer, ales, and milk can be prepared and kept indefinitely without any regard to outside conditions of the atmosphere. One of the practical and valuable features of the process is that all the liquids named can be put up in siphons and supplied in this most convenient form to the consumer. The latter can then use any desired quantity of the contents of the siphon and keep the remainder intact for future use. For invalids and those who do not care to use alcoholic liquors the process permits the pure expressed juice of the fruit being preserved with its natural flavor and bouquet. The value of pure carbonated milk for ready consumption will also be appreciated by physicians. Just as mineral waters are delivered to the consumer by the bottler or grocer, so can siphons of assorted liquors, such as beer and ale, be delivered in a similar manner. The fact that the drawing of corks and consequent exposure of the liquid to deterioration is done away with makes the new process as economical as it is practical and convenient."

In the cellar beneath the company's offices was shown the machinery used in the two processes of exhausting the air from the liquid and replacing it with the carbonic acid gas. The invention, Mr. Murdoch said, would also prove a very effective and simple arrangement for drawing ale or beer from the cellar to the bar, and will insure the beer being always in proper condition.

